# Potential Use of *Bacillus coagulans* in the Food Industry

**DOI:** 10.3390/foods7060092

**Published:** 2018-06-13

**Authors:** Gözde Konuray, Zerrin Erginkaya

**Affiliations:** Department of Food Engineering, Cukurova University, Adana 01330, Turkey; zerriner@cu.edu.tr

**Keywords:** *Bacillus coagulans*, probiotic, microbial enzyme

## Abstract

Probiotic microorganisms are generally considered to beneficially affect host health when used in adequate amounts. Although generally used in dairy products, they are also widely used in various commercial food products such as fermented meats, cereals, baby foods, fruit juices, and ice creams. Among lactic acid bacteria, *Lactobacillus* and *Bifidobacterium* are the most commonly used bacteria in probiotic foods, but they are not resistant to heat treatment. Probiotic food diversity is expected to be greater with the use of probiotics, which are resistant to heat treatment and gastrointestinal system conditions. *Bacillus coagulans* (*B. coagulans*) has recently attracted the attention of researchers and food manufacturers, as it exhibits characteristics of both the *Bacillus* and *Lactobacillus* genera. *B. coagulans* is a spore-forming bacterium which is resistant to high temperatures with its probiotic activity. In addition, a large number of studies have been carried out on the low-cost microbial production of industrially valuable products such as lactic acid and various enzymes of *B. coagulans* which have been used in food production. In this review, the importance of *B. coagulans* in food industry is discussed. Moreover, some studies on *B. coagulans* products and the use of *B. coagulans* as a probiotic in food products are summarized.

## 1. Introduction

Nowadays, the interest in probiotic foods is increasing due to the growing consumer demand for safe and functional foods with health-promoting properties and high nutritional value [1]. Probiotics are defined as “live microorganisms that, when administered in adequate amounts, confer a health benefit on the host” [2]. In order to obtain benefits, probiotic products should contain at least 10^7^–10^9^ cfu/g probiotic microorganism and should survive until the end of shelf life [3]. Probiotic microorganisms, which are naturally found in intestinal microbiota, could protect humans from diseases, modulate and strengthen the immune system, prevent tooth decay, have anticarcinogenic properties, and be effective against coronary heart disease [4,5]. Probiotic microorganisms can produce organic acids (such as lactic and acetic acid), hydrogen peroxide, and bacteriocin [5]. Probiotics have several mechanisms to inhibit pathogen microorganisms. The primary mechanisms are as follows: (1) the lowering of the pH of food through lactic acid production; (2) the production of antimicrobial substances such as microcin, hydrogen peroxide, and compounds like free radicals; (3) competition for food resources by attaching to receptors; and (4) stimulation of the production of secretory IgA (Immunoglobulin A) by the formation of protective mucin (parent substance of the mucus composed of tissue of epithelial or connective origin and a mixture of glycoprotein and mucoprotein) [5]. 

There are two basic forms of probiotic microorganisms used in foods: the vegetative form and the spore form. The vegetative form is more susceptible to high temperatures, moisture, acidity, shelf life of food, and negative environmental conditions during the manufacture of food than the spore form. However, some probiotic microorganisms do not have spore forms [4]. Fermentation conditions, freezing, thawing, drying, cell protection additives, rehydration of dried probiotics, and microencapsulation applications are factors that affect the survival of probiotic microorganisms during probiotic food production. Food compounds, food additives, oxygen content, redox potential, moisture content/water activity, storage temperature, pH and titration acidity, and packaging conditions are factors that also affect survival of probiotic microorganisms during storage [6]. Gastrointestinal system conditions and stress factors could cause significant loss of viable probiotic cells [7]. 

Lactic acid bacteria (LAB; for example, *Lactobacillus* and *Bifidobacterium* and some *Saccharomyces* species) are the microorganisms most commonly used in probiotic food production [8,9,10,11]. However, these microorganisms cannot survive heat treatment, for which the cold spot temperature is approximately 75 °C [8,10]. Heat treatment is not applicable for most probiotic foods that contain commercial probiotic microorganisms due to their sensitivity to heat. Nevertheless, it has been stated that this restriction could be overcome by the usage of spore-forming probiotic microorganisms. It is known that some non-pathogenic *Bacillus* species, which are not as well-known as LAB and yeasts, are being used as probiotics [12]. The survival and stability of these bacteria have considerably improved compared to others through their spore-forming abilities. They are identified as an ideal choice in order to development of functional foods by protecting their vitality in high-temperature applications [13,14].

*Bacillus coagulans* (*B. coagulans*) was firstly isolated from spoiled milk [6]. In 1933, it was identified as *Lactobacillus sporogenes* by Horowitz-Wlassowa and Nowotelnow. Afterwards, it was classified as *B. coagulans* [15].

*B. coagulans* is a gram-positive, facultative anaerobic, nonpathogenic, spore-forming, lactic acid-producing bacteria [4]. It is resistant to heat; the optimum growth temperature for *B. coagulans* is 35 to 50 °C and the optimum growth pH is 5.5 to 6.5 [4,15]. It has the characteristics of microorganisms used as probiotics [15]. Some strains of *B. coagulans* have been reported as facultative anaerobe, thermophile bacteria able to grow at pH 6.2, 60–65 °C [6,16]. Although *B. coagulans* produces acid, it does not produce gas from maltose, raffinose, mannitol, and sucrose fermentation. It was reported that *B. coagulans* causes deterioration in dairy, fruit, and vegetable products due to acid production. In addition to lactic acid production, some strains also produce thermostable α-amylase [4,17]. For this reason, *B. coagulans* is important from an industrial point of view. *B. coagulans* spores are terminal, while spores of other species are central or subterminal. Furthermore, it differs from other *Bacillus* species due to the absence of cytochrome-C oxidase, and it does not reduce nitrate to nitrite [4]. It was reported that *B. coagulans* could grow at pH 4.5 at 65 °C and was isolated from products containing milk and carbohydrate [18].

*B. coagulans* has been reported as safe by the US Food and Drug Administration (FDA) and the European Union Food Safety Authority (EFSA) and is on the Generally Recognized As Safe (GRAS) and Qualified Presumption of Safety (QPS) list [19]. In addition, it was reported that genome sequencing can provide information about the overall characterization of the bacterium, for example with respect to its safety as a food supplement [20]. The *B. coagulans* GBI-30, 6086 genome was investigated, and it was found that it did not contain any hazardous genes [21]. Some of the non-pathogenic strains among the 100 known *Bacillus* spp., including *B. coagulans* and *Bacillus subtilis* var. *natto*, were stated as safe for human consumption [22,23].

## 2. Probiotic Activity of *B. coagulans*

Heat-treated food products are generally not used for probiotic purposes because of the factors affecting their viability and stability [15]. In order to obviate this difficulty, *B. coagulans*, *Bacillus racemilacticus*, and *Bacillus laevolacticus* as well as the *Sporolactobacillus* genus could be used as probiotics due to their heat-resistant spore forms [12,15]. Although there are limited research studies on the use of *Bacillus* spp. in human nutrition, many food products containing *B. coagulans* have been sold in various countries (Table 1). Traditionally, probiotic microorganisms have been used as freeze-dried in probiotic food supplements, in dairy products such as yogurt, and in fermented beverages [24,25,26]. The viability and stability of these bacteria improved considerably compared to others by means of spore formation. It is stated that they are an ideal choice for the development of cereal-based functional products because they can maintain their viability in heat-treated processes such as baking and boiling. In addition, the spores gain a stable state during the food storage [13].

The survival rates of *Lactobacillus* strains are highly affected by the production process, storage, and transportation of food. It is reported that some strains of *B. coagulans* are better able to survive in high-temperature heat treatment and stomach conditions than other commercial probiotic microorganisms. It is suggested that strains which have these properties are likely to survive better in the digestive tract [29]. 

*B. coagulans* GBI-30, 6086 is a commercial probiotic mixture also known as GanedenBC^30^ [13,20]. Many research studies have been conducted and have reported the beneficial effects of *B. coagulans* GBI-30, 6086 on human and animal health [30,31,32]. It has been reported as safe by EFSA, and included in the GRAS and QPS list. It is available in various probiotic foods in markets [13,20]. 

In a study by Hyronimus et al. (2000) [12], the influence of pH value and bile salt concentration of culture media on LAB growth was observed. It was reported that *B. coagulans* was resistant to 0.3% bile salt concentration. In a study by Bora et al. (2009) [33], the effect of hygroscopicity, pH stability, and additives (lactose monohydrate, dibasic calcium phosphate dihydrate, microcrystalline cellulose, and corn starch) on *B. coagulans* spores were determined. The spores were found to be hygroscopic; maximum stability was screened at pH 6.8. There was no difference observed with additive addition. 

In a study by Lee et al. (2017) [34], the probiotic activity of *Bacillus* spp. isolated from traditional Korean soy sauce was evaluated. Three of the isolates (MKSK-E1, MKSK-J1 and MKSK-M1) were found to be quite resistant to gastrointestinal tract conditions and showed antibacterial activity against *B. cereus*, *Listeria monocytogenes* (*L. monocytogenes*), *Staphylococcus aureus* (*S. aureus*), and *Escherichia coli* (*E. coli*)*.* It was stated that these isolates could be used as probiotics in functional foods and animal feeds by evaluating antibiotic resistance, biogenic amine production, and hemolytic properties. 

In a number of studies, antimicrobial activity of bacteriocin produced from *B. coagulans* was evaluated. Abada (2008) [35], reported that bacteriocin produced from *B. coagulans* had an inhibitory effect against *E. coli* (NCTC-10418), *Pseudomonas aeruginosa* (NCIB-9016), *Klebsiella pneumoniae* (NCIB-9111), *B. subtilis* (NCTC-6346), *Staphylococcus aureus* (*S. aureus*) (NCTC7447), and *Candida albicans* (CBS-562). Natarajaseenivasan et al. (2015) [36] reported on the antimicrobial effect of bacteriocin produced from *Bacillus coagulans* BDU3, which was isolated from traditional fermented fish. It was stated that it has an inhibitory effect on food-borne pathogens such as *B. cereus* MTCC 430, *S. aureus* MTCC 3160, *Enterococcus* sp. MTCC 9728, *Lactobacillus* sp. MTCC 10093, and *Micrococcus luteus* MTCC 106. In addition, it was found that *Bacillus coagulans* BDU3 survived in pH 2.0 and 0.2% bile salt concentration. Senna and Lathrop (2017) [37] reported that *B. coagulans* had an antifungal effect on *Botrytis cinereal*, *Fusarium pallidoroseum*, and *Fusarium moniliforme*. Donskey et al. (2001) [38], researched the antimicrobial effect of *B. coagulans* on vancomycin-resistant enterococcus found in rat intestine. Rats were fed with *B. coagulans* at a concentration of 10^7^ cfu/g for 4 days. *B. coagulans* showed an inhibitory effect on vancomycin-resistant enterococci count.

According to the Jafari et al. (2016) [39], viability and growth of *B. coagulans* spores in sausages were affected by formulation, chopping, and surfactant. In a study by Jafari et al. (2017) [40], *B. subtilis* var. *natto* ATCC 15245 and *Bacillus coagulans* ATCC 31284 spores were inoculated in sausages. Different household-type cooking methods (boiling, microwave cooking, and deep frying) and cold storage were applied to sausages and survival rate of spores were evaluated. They reported that after heat treatment, the *B. coagulans* count was sufficient to define the sausages as probiotic. In another study by Taguchi (1986) [41], it is reported that spore count was affected by sausage formulation and cooking method. Fares et al. (2015) [13] used *B. coagulans* GBI-30, 6086 in order to produce functional pasta. After the production processes and cooking (5–7 min) were applied, the *B. coagulans* count was 9 log cfu/100g. They reported that this concentration of probiotic bacteria containing in pasta was enough to show a beneficial effect for consumers, with high nutritional value and sensorial properties. 

Generally, probiotic *Bacillus*-containing foods and feeds are used as food supplements for humans, growth enhancers for animals, and growth regulators or protectors against diseases in aquaculture [26]. It is reported that regular consumption of probiotic microorganism-containing products strengthens the immune system, exhibits an anti-allergy effect, reduces cancer risk, lowers cholesterol, prevents digestive problems, and reduces gastrointestinal system infections [3]. It is reported that if *B. clausii*, *B. coagulans*, and *B. subtilis*-containing probiotic preparations are consumed on a regular basis, gastrointestinal disorders such as childhood diarrhea can be prevented [42,43,44] and the duration of respiratory tract infections in children is reduced [45]. These preparations can also be used in the treatment of symptoms associated with irritable bowel syndrome [46]. Endres et al. (2009) [29] performed a bacterial reverse mutation test, chromosomal abnormality test, micronucleus test, acute and 90-day sub-chronic recurrent toxicity test, and an acute eye and skin irritation test in order to determine toxicological properties of *B. coagulans*. It was reported that *B. coagulans* was found suitable for human consumption.

In the case of *B. coagulans* consumed as a feed additive, it prevents *E. coli* and *Staphylococcus* infections in gastrointestinal system and significantly improves the development of animals (such as poultry, pigs, etc.) by increasing digestibility [4].

## 3. Products of *B. coagulans*

In recent years, biological production of many metabolites (such as ethanol, lactic acid, fumaric acid, xylonix acid and other important products) has attracted greater attention as compared to chemical production with petroleum materials [47]. Various substances produced by *B. coagulans* are shown in Table 2.

Among these metabolites, lactic acid is an important product based on its high yield. Due to the demand for biodegradable and biocompatible materials, interest in lactic acid is increasing day by day [47]. *B. coagulans* is an ideal microorganism in industrial lactic acid production due to its ability to ferment glucose and xylose to lactic acid in anaerobic conditions at temperatures under 50 °C [68,69,70]. In many research studies, lactic acid production from *B. coagulans* was carried out using sugarcane pulp [49], sorghum water [50,51], coffee extract [52], wheat straw [53], corn cob [54,55], lignocellulosic hydrolysate [71], and corn flour [56] as substrates.

β-galactosidase is used for lactose hydrolysis in the food industry. It is found commonly in nature, and isolated from animals, plants, and microorganisms. It is a widely used enzyme in the food and pharmaceutical industry. Production of this enzyme from microorganisms allows for higher yields and a greater technological advantage compared to other sources. β-galactosidase also could be produced by several yeast (*Kluyveromyces lactis* and *Kluyveromyces marxianus*), mold (*Aspergillus niger* and *Aspergillus oryzae*), and bacteria (*B. circulans*, *E. coli*, *Lactobacillus bulgaricus*, *Lactobacillus thermophile*, *Geobacillus stearothermophilus*), with the exception of *B. coagulans* [72,73]. α-galactosidase is an important enzyme in the food industry, and is used many applications, especially in sugar production, biotechnology, and medicine. The most important use in the food industry is sugar production. α-galactosidase can also facilitate the digestion of legumes, such as soybean, through removing galactosides such as raffinose and stachyose from their structures and enhancing the gelation capacities of galactomannans [74]. Several studies have reported on α-galactosidase produced by *B. coagulans* [62,63].

It has been reported that *B. coagulans* has proteolytic activity [75,76]. Amylases are important hydrolase enzymes that have been used for many years. α-amylases are demanded in high amounts due to their wide industrial applications. α-amylases can be produced from plants and microorganisms. Being a cheap source of α-amylase production, microbial production is attracting attention as compared to others [77,78]. In many studies, α-amylase production was carried out using *B. coagulans* [64,78]. There is a great interest in the production of lipases from microorganisms, despite the presence of lipase in many animals, plants, and microorganisms. It is widely used in the food industry as well as in the pharmaceutical, textile, and cosmetic industry [79,80]. Among bacteria, a few *Bacillus* species are identified as producing lipase [81]. In previous studies, lipase production from *B. coagulans* was determined [65,66,67,81].

Xylanase is widely used in clarification process of fruit juices and wines. Several studies have been carried out to produce xylanase by *B. coagulans* [82,83]. 

## 4. Conclusions

Consumer interest in healthier and more functional food is increasing due to changing consumption habits and increasing interest in food and health. In addition to supporting the clinically beneficial effects of probiotic microorganisms on health, the formulation of probiotic food products has great importance for consumers, industry, and research centers which are interested in the subject. Heat-resistance of probiotic *Bacillus* spp. spore forms can provide an advantage for heat-treated probiotic foods. *B. coagulans* is attracting interest due to its resistance to strong gastric acid and high temperatures, and it is more resistant to antibiotics than other LAB. Moreover, the products, which are used in food industry and could be produced by *B. coagulans*, are gaining attention due to their low cost and as an alternative to other chemical sources.

## Figures and Tables

**Table 1:** Probiotic food supplements containing *Bacillus coagulans.*

Strain	Supplement	Reference
*Bacillus coagulans* 15B	Nutrition essentials Probiotic	[27]
*B. coagulans* and *Bacillus subtilis* (*B. subtilis*)	NutriCommit	[27]
*B. coagulans* and *Saccharomyces boulardii*	Flora3	[27]
*B. coagulans*	THORNE	[27]
*B. coagulans*	Sunny Green Cleansing Green	[27]
*Bacillus indicus* HU36, *B. coagulans*, *Bacillus clausii* (*B. clausii*), *Bacillus subtilis* HU58	Just Thrive	[27]
*Bacillus indicus*, *B. subtilis*, *B. coagulans*, *Bacillus licheniformis*, *B. clausii*	MegaSporeBiotic	[27]
*B. coagulans*	Sustenex	[26]
*B. coagulans*	Neolactoflorene	[26]
*B. coagulans*	GanedenBC30	[28]

**Table 2:** Substances produced by *B. coagulans.*

Strain	Substrate	Product	Reference
*Bacillus coagulans* DSM 2314	Wheat straw	Lactic acid	[48]
*Bacillus coagulans* DSM2314	Sugarcane bagasse	Lactic acid	[49]
*B. coagulans*	Sorghum water	Lactic acid	[50,51]
*B. coagulans*	Coffee extract	Lactic acid	[52]
*Bacillus coagulans* IPE 22	Wheat straw	Lactic acid	[53]
*Bacillus coagulans* LA 204	Corn stover	Lactic acid	[54]
*B. coagulans*	Corn stover	Lactic acid	[55]
*Bacillus coagulans* HL-5	Corn flour	Lactic acid	[56]
*Bacillus coagulans* TB/04	Medium	Lactic acid	[57]
*Bacillus coagulans* PS5	Medium	Lactic acid	[58]
*Bacillus coagulans* arr4	Granulated sugar and yeast extract	Lactic acid	[59]
*Bacillus coagulans* JI12	Oil palm empty fruit bunch	Lactic acid	[60]
*Bacillus coagulans* RCS3	Medium	β-galactosidase	[61]
*Bacillus coagulans* KM-1	Fermented soybean	α-galactosidase	[62]
*Bacillus coagulans* BL174	Medium	α-galactosidase	[63]
*Bacillus coagulans* B49	Wheat bran	α-amylase	[64]
*Bacillus coagulans* BL174	Medium	Lipase	[63]
*Bacillus coagulans* ZJU318	Medium	Lipase	[65]
*B. coagulans*	Melon wastes	Lipase	[66]
*Bacillus coagulans* VKl1	Coconut oil cake	Lipase	[67]
